# Cerebro-Spinal Involvement in Hereditary Syphilis

**Published:** 1920-03-20

**Authors:** 


### Cerebro-spinal Involvement in Hereditary Syphilis.

To recall the pathological changes observable in the cerebro-spinal fluid of early syphilitics, and the indications which these changes give of early involvement of the central nervous system, as pointed out in the report of Fildes and Parnell, we refer the reader to a review recently given in these pages (February 28, p. 507).

Another Contribution.

Another contribution to this subject now comes from the paper by Dr. Jeans, of St. Louis, which was read before the American Pedriatic Society, and from which we abstract the purely medical bearing. He found that about 30 per cent. of syphilitic infants had changes in the cerebro-spinal fluid which, according to modern conceptions, were of such a character and degree as to make the diagnosis of syphilitic involvement of the nervous system seem quite certain. Obviously, many of these cases had no clinical manifestations of nervous disease, but the result of anti-syphilitic treatment was suggestive. Exactly as intravenous or intramuscular inoculation of the specific compounds will produce a negative Wassermann reaction, so was this treatment found to clear up the abnormal serological reaction and cytology in the infants' cerebro-spinal fluid. Older children showed a very similar percentage among those examined, but in some of these the signs of early neurosyphilis were present.

The Effect of Treatment.

In both age classes the great point to be noted from Dr. Jeans' observations is that prolonged anti-syphilitic treatment will eventually result in a negative blood test, while the cerebro-spinal fluid may or may not continue to show typical changes. When, in his cases, intraspinal treatment was applied, however, there was rapid disappearance of cerebro-spinal fluid abnormalities.

The conclusions which the author draws from his series are in all probability fully justified. Added to those from Haslar, which we have already quoted, these findings insist that in justice to patients the state of the thecal fluid must be examined in every case as the only true index of early neurosyphilis. No greater error could be committed than to allow a patient's discharge after being treated to clinical recovery and a negative blood reaction without first knowing through the agency of lumbar puncture and a laboratory examination of the fluid whether signs of neurosyphilis still persist.

